# Combination of neoadjuvant and adjuvant chemotherapy with FOLFOX compared with adjuvant chemotherapy in management of locally advanced rectal cancers: a randomized trial of a promising therapeutic approach

**DOI:** 10.1186/s12885-024-12634-7

**Published:** 2024-07-18

**Authors:** Mandana Biniaz, Arash Moradi, Manouchehr Ghorbanpour Basit, Abdol-Azim Seddighi Pashaki, Arash Dehghan, Kamal Mohammadian

**Affiliations:** 1 Department of Radiation Oncology, Faculty of Medicine, Hamadan University of Medical Sciences, Hamadan, Iran; 2 Department of Molecular Medicine, Medical Biotechnology Institute, National Institute of Genetic Engineering and Biotechnology, Tehran, Iran; 3 Department of Surgery, Faculty of Medicine, Hamadan University of Medical Sciences, Hamadan, Iran; 4 Cancer Research Centre, Hamadan University of Medical Sciences, Hamadan, Iran; 5 Department of Pathology, Faculty of Medicine, Hamadan University of Medical Sciences, Hamadan, Iran; 6 Cancer Research Centre, Hamadan University of Medical Sciences, Hamadan, Iran

**Keywords:** Neoadjuvant therapy, Disease-free survival, Follow-up studies, FOLFOX, LARC

## Abstract

**Background:**

Colorectal cancer (CRC) is a significant malignancy with widespread implications. Despite progress in surgical interventions for rectal cancer, improvements in overall prognosis remain disproportionate. Standard preoperative chemoradiation, while established as the standard treatment for the majority of rectal cancers, exhibits limited effectiveness in enhancing disease-free survival (DFS) and mitigating distant metastases, particularly in cases of locally advanced rectal cancer (LARC).

**Methods:**

This randomised clinical trial assessed 286 patients with LARC in two paralleled groups. Group A underwent six courses of neoadjuvant MFOLFOX chemotherapy, chemoradiation, surgery, and six adjuvant chemotherapy cycles. Group B received concurrent chemoradiation, surgery, and twelve adjuvant chemotherapy cycles. Patient evaluations were achieved at multiple stages of treatment and follow-up.

**Results:**

Group A had significantly lower local recurrence (11.64%) than Group B (21.74%, *P* = 0.025). The distant metastasis rate in Group A (8.90%) was lower than in Group B (20.29%) but was not significant (*p* = 0.143). More patients in Group A experienced downstaging (80.82% vs. 60.87%, *p* < 0.001). Specifically, 72.60% demonstrated downstaging of tumour invasion and 54.79% downstaging of lymph node involvement, compared to 57.25% and 41.30% in Group B (*p* = 0.009 and *p* = 0.025, respectively) as well as higher pCR rate (26.03% vs. 15.25%, *p* = 0.030) and three-year DFS rate (82.19% vs. 71.01%, *p* = 0.035) in group A compare to group B.

**Conclusion:**

This innovative strategy for LARC showed promising results with lower local recurrence and higher rates of downstaging and pCR. Treatment side effects were similar in both groups but less frequent in Group A. Anaemia was the most common haematological side effect (A: 58%, B: 68%), and peripheral sensory neuropathy was the most common non-haematological complication (A: 63%, B: 64%). These findings suggest this regimen could be a valuable therapeutic approach for LARC.

**Trial Registration:**

This trial was registered on 2023–12-08 within the IRCT.IR database under the number IRCT20210308050628N1.

## Introduction

Recent data from the Global Cancer Observatory highlights colorectal cancer (CRC) as the third most-diagnosed cancer and the second leading cause of cancer death globally in 2023 [1]. CRC represents 10% of all cancer diagnoses and 9% of cancer deaths, with an estimated 1.9 million new cases and 0.9 million deaths annually. Despite a 2% decrease in mortality over the past two decades, there is a concerning 3.9% increase in CRC incidence among individuals under 50 [2]. Geographic disparities in CRC incidence and mortality are significant, reflecting differences in risk factors, screening, and healthcare access. Developed regions report higher CRC rates, ranking it as the second or third most common cancer among both genders [3].

Conversely, developing countries, primarily in Africa and Asia, exhibit lower CRC rates, ranking lower among the top ten cancers. Nonetheless, many low and middle-income nations’ records indicate increased CRC incidence and mortality rates, while high-income countries are experiencing a decreasing or stabilising trend [4] Socioeconomic development, lifestyle changes, and improvements in the health system could influence this trend.

Rectal cancer is a common malignancy that requires multimodal treatment to achieve local control and prevent distant recurrence. However, the treatment results of locally advanced colorectal cancers, especially tumours that invade adjacent structures or have extensive nodal involvement, are still disappointing despite the advances in surgical techniques [5]. About 30% of patients with locally advanced rectal cancer (LARC) develop distant metastases, the leading cause of mortality. Therefore, systemic therapy has gained importance in managing LARC, as it can target micrometastases, decrease distant metastases, and improve survival outcomes. The current standard of care for LARC is preoperative chemoradiotherapy followed by surgery plus/minus adjuvant chemotherapy [6–8]. However, this sequence has some limitations, such as poor compliance, delayed surgery, and reduced efficacy of adjuvant chemotherapy due to postoperative complications and impaired drug delivery to the tumour blood and microenvironment [9]. Previous studies have demonstrated that the neoadjuvant combination of chemotherapy and radiotherapy increases local control and reduces the side effects of the treatment more than postoperative chemotherapy and radiotherapy in rectal cancers [10, 11]. Besides, more pathologic complete response and tumour downstaging have been seen following neoadjuvant chemoradiation, which causes a reduction in local recurrence and more sphincter-saving surgery [12]. The National Comprehensive Cancer Network (NCCN) guidelines have recently proposed an alternative strategy to overcome these challenges: administering neoadjuvant chemotherapy before chemoradiotherapy and surgery [13]. This approach, known as total neoadjuvant therapy (TNT), has several potential advantages over the conventional sequence, such as better tolerance, higher drug concentration in the tumour microenvironment before surgery, increased tumour shrinkage and radiosensitisation, and higher rates of pathological complete response and organ preservation [14].

Consequently, TNT may improve patients’ quality of life and prognosis with LARC. However, the possibility of distant recurrence is still present, probably because of the problems during the surgery, tumour manipulation, and the likelihood of micrometastasis creation at the time of operation. Therefore, post-surgery chemotherapy may help eliminate these micrometastases.

**Fig. 1:**
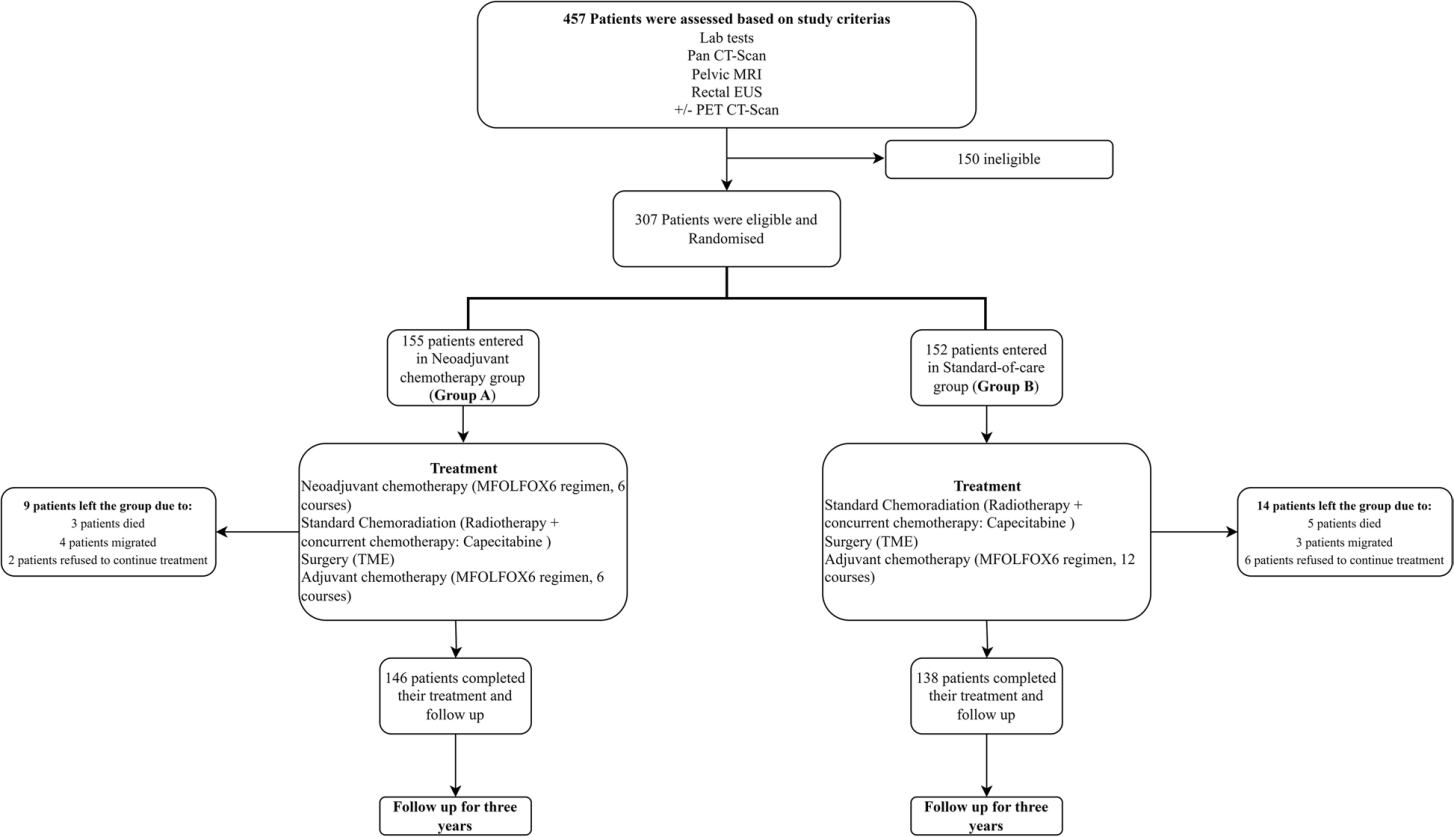
Enrolment, Treatment, and Follow-up

Our objective was to assess the efficacy of a neoadjuvant sandwich treatment protocol for locally advanced rectal cancer. This approach integrates induction chemotherapy, concurrent chemoradiation therapy, and consolidation chemotherapy after surgery, compared with the standard treatment that involves chemoradiation, followed by surgery and chemotherapy (Fig. 1). Our study aimed to determine the complete pathological response, primary tumour and lymph nodes downstage, and three-year disease-free survival.

## Methods

### Eligibility and pre-treatment evaluation

Initially, we evaluated the transparency indicators, including the completeness of reporting as per the CONSORT statement and the extent of data sharing [15]. This clinical trial aims to investigate the effects of neoadjuvant chemotherapy on locally advanced rectal cancers through a two-arm parallel group randomised study. The study comprises individuals between the ages of 18 and 75 years with confirmed rectal adenocarcinoma by histopathology or Immunohistochemistry and located 2 to 15 cm from the anal verge. Patients with locally advanced rectal cancer (T3-T4 or N +) were identified through magnetic resonance imaging (MRI) or endorectal ultrasound (EUS). They were required to have no evidence of distant metastasis on computed tomography (CT) or positron emission tomography plus computed tomography (PET-CT) scans. In addition, participants had to meet specific criteria for Eastern Cooperative Oncology Group (ECOG) performance status and hematologic, liver, and renal functions. All participants underwent a chest, abdomen, and pelvis CT scan.

Serum carcinoembryonic antigen (CEA) and CA19-9 levels were measured. Patients with recurrent rectal cancer, prior pelvic radiation, or previous history of other malignancies were excluded. The American Joint Committee on Cancer (7th edition) TNM system was used for staging. Following initial assessments and identification of eligible patients for study inclusion, these patients were assigned to intervention or control groups based on their admission numbers (1:1). Individuals with odd admission numbers were allocated to the intervention group. In contrast, those with even numbers were assigned to the control group. The surgeons, pathologists, and data analysis team are unaware of the treatment methods and treatment groups of patients. In contrast, the oncologists and the data collection and registry team knew the treatment modalities and treatment groups. This study was conducted as a phase three randomised clinical trial. The therapeutic interventions, including radiation therapy and chemotherapy, were performed at the cancer centre. In addition, supplementary diagnostic and therapeutic interventions such as surgery, pathology, laboratory tests, endoscopic ultrasound (EUS), magnetic resonance imaging (MRI), computed tomography (CT) scans, and positron emission tomography (PET) CT scans were performed at various academic, private, public, and charity centres. Trained and specialised colleagues within the study team carried out these measures. One significant limitation of this investigation is the need for a centralised review of study results at the participating centres. The decentralised approach may have introduced variability in data collection, interpretation, and reporting, potentially affecting the strength and consistency of our findings. The sample size was determined based on the guidelines for determining sample size in the health sciences [16]. The Hamadan University of Medical Sciences ethics committee approved the study protocol and followed the principles outlined in the Declaration of Helsinki (IR.UMSHA.REC.1401.768), with informed consent obtained from all participants before treatment initiation. This trial is registered with IRCT.IR, number IRCT20210308050628N1.

### Chemotherapy

Three hundred and seven patients with locally advanced rectal cancer were enrolled in two therapeutic lines, among which one hundred fifty-five were placed in group A (Neoadjuvant chemotherapy group), of which 146 patients completed their treatment and follow-up. They were treated first with six cycles of chemotherapy, including oxaliplatin 85 mg/m2 on day 1, Calcium Folinate 200 mg/m2 per day on day 1 and 2 followed by 5-FU bolus 5-fluorouracil (5FU) 400 mg/m2 on day 1 and 1200 mg/m2 per day for two consecutive days every two weeks (the MFOLFOX6 regimen), followed by standard chemoradiation therapy, including radiotherapy with concurrent capecitabine 800–1000 mg/m2 BID (twice-daily in radiotherapy days) followed by surgery. Six to eight weeks after surgery, these patients received six more chemotherapy cycles (the MFOLFOX6 regimen). Conversely, one hundred fifty-two patients were placed in group B (Standard-of-care group), and one hundred thirty-eight patients completed their treatment period. They were treated with standard concurrent chemoradiation therapy (Radiation therapy plus capecitabine) followed by surgery. They received 12 cycles of MFOLFOX6 chemotherapy six to eight weeks after surgery. We used G-CSF after each cycle of chemotherapy.

### Radiotherapy

In this study, two groups were subjected to radiation therapy using a CT simulator and a linear accelerator employing the 3D-Conformal therapy method, using high-energy photons (18MV). The radiation dose delivered was 50.4 Gy over approximately six weeks (28 fractions), with a daily fraction of 180cGy. The treatment planning and dose calculation were done using the Monaco treatment-planning system with a Monte Carlo algorithm.

The simulations were conducted with patients in a prone position and with a full bladder, and the CT-based simulation with a slice thickness of 3 mm was performed. Target volumes were determined following the International Commission on Radiation Units and Measurements Reports 50 ICRU guidelines. The gross tumour volume (GTV) was delineated, and any enlarged lymph nodes observed on a CT scan or magnetic resonance imaging (MRI) were marked.

The clinical target volume (CTV) encompassed the GTV with a radial margin of 2 cm for the true pelvis and a craniocaudal margin of at least 3 cm. It included lymphatic drainage areas such as the sacral, iliac lymph nodes, obturator lymph drainage, and the true pelvis internal iliac lymph drainage area. The prescribed irradiation dose for the treatment involves delivering 45 Gy to the pelvis in 25 fractions. This was achieved by administering a daily dose of 1.8 Gy five days a week. The irradiation technique employed was the Box technique, utilising four fields daily. A boost dose of 5.4 Gy was also administered to the gross tumour, resulting in a total dose of 50.4 Gy. The radiation was delivered using 18MV photons and the 3D/CRT (three-dimensional conformal radiation therapy) method. In the treatment planning process, comprehensive attention was directed towards the dosage prescription and limitations concerning delicate organs, notably the small bowel and bladder. These organs were considered to ensure that the radiation dose delivered to them remained within acceptable limits, thereby minimising the potential for adverse effects. In addition, the concurrent administration of capecitabine at a dose of 800–1000 mg/m2 BID (twice daily on radiotherapy days) was employed to complement the treatment.

### Surgery

Surgical intervention, adhering to the principles of total mesorectal excision (TME), was meticulously scheduled to occur within a 6 to 8-week window following the culmination of radiation therapy. This interval allowed for optimal healthy tissue recovery and response evaluation post-radiation. During the procedure, patients underwent a comprehensive radical rectal resection, which is a cornerstone technique in rectal cancer surgery aimed at removing the cancerous tissue along with the associated lymphovascular structures within the mesorectal fascia. The TME approach minimises local recurrence rates and enhances overall surgical outcomes.

### Treatment evaluation

Four weeks following the conclusion of chemoradiation, a comprehensive evaluation was carried out, incorporating a thorough physical examination, digital rectal examination, assessment of serum levels of carcinoembryonic antigen (CEA) and carbohydrate antigen 19–9 (CA19-9), computed tomography (CT) scanning of the chest, abdomen, and pelvis, endoscopic ultrasonography, and pelvic magnetic resonance imaging (MRI). Assessments of adverse events were conducted weekly in the clinical setting. The patient’s laboratory findings and unfavourable occurrences were monitored throughout treatment. The entailed monitoring had been achieved before and after each cycle of neoadjuvant chemotherapy, every week during chemoradiotherapy, before and after the surgical procedure, and preceding all rounds of adjuvant chemotherapy. The laboratory examinations encompassed evaluating serum levels of aspartate transaminase, alanine transaminase, total and direct bilirubin, and serum creatinine before administering neoadjuvant and adjuvant chemotherapy. Any untoward events linked to preoperative and adjuvant therapies were evaluated and graded using the Common Terminology Criteria for Adverse Events (CTCAE), version 4. A severe adverse event was defined as an event leading to death, sustained or considerable temporary disability or incapacity, a congenital anomaly, congenital disability, or abortion; posing a life-threatening risk; necessitating hospital admission or prolongation of existing hospitalisation; or any clinically significant incidence affecting the safety of the participants.

### Pathologic examination

Specimens, comprising initial biopsies and subsequent surgical samples, underwent evaluation by proficient pathologists. Only adenocarcinomas validated by the pathology, encompassing well, moderately, and poorly differentiated types, were considered for inclusion in the study. Suspicious findings were subjected to reassessment utilising the immunohistochemistry (IHC) methodology. Confirmed items with IHC entered the study.

### Endpoints

The primary endpoints of this study were to assess the rate of pathologic complete response (pCR), which was defined as the absence of any remaining tumour in the original location or dissected lymph nodes, and the three-year disease-free survival (3y-DFS), which was defined as the absence of tumour in clinical examinations and Para clinical modalities from the completion of treatment (adjuvant chemotherapy) until three years later. Additional critical endpoints included tumour down-staging (T), lymph node down-staging (N), and local and distant recurrence incidence.

### Follow-up

After the treatment, patients underwent stringent monitoring involving regular physical examination, complete blood count (CBC) assessments, renal and hepatic function tests, and determination of serum levels of carcinoembryonic antigen (CEA) and carbohydrate antigen 19–9 (CA19-9) every three months. Additionally, chest and abdominopelvic CT scanning was conducted every 6–12 months, and colonoscopy was performed annually for three years.

### Statistical analysis

The appropriate sample size for this study based on our population was determined using the Cochran formula with a precision level of ± 5%, a confidence level of 95%, and an estimated proportion of 0.5. The study’s findings were delineated by representing mean values and their corresponding standard deviations (SD). The data was analysed using GraphPad Prism version 9.3.1 for Windows, a software developed by GraphPad Software in San Diego, California, USA, lauded for its precision and dependability in data analysis. Fisher’s exact test, a statistical test renowned for its applicability in small sample sizes, was employed to ascertain the statistical significance of disparities between groups. A p-value less than 0.05 was deemed to indicate statistical significance.

## Results

From January 2015 to November 2019, 284 patients who participated in the study completed their treatment and follow-up. The appropriate sample size was at least 171 patients, given the population size of 307 patients, precision level of ± 5%, confidence level of 95%, and estimated proportion of 0.5. Their clinicopathological features are listed in Table 1. A detailed analysis of the clinicopathological characteristics of Group A (Neoadjuvant chemotherapy) and Group B (Standard-of-care) revealed multiple differences. Firstly, the age distribution in Group A was 58.29 ± 8.496 years, and in Group B was 59.72 ± 9.137 years.

**Table 1:** Baseline demographic and clinical characteristics in the intention-to-treat population

Variable		Group A (Neoadjuvant chemotherapy) (*n* = 146)	Group B (Standard-of-care) (*n* = 138)	*P* Value
**Age at randomisation (years)**	Mean ± SD	58.29 ± 8.496	59.72 ± 9.137	NA
	Range	41–74	30–72	NA
**Sex**	Female	65 (44.52%)	43 (31.16%)	NS
	Male	81 (55.48%)	95 (68.84%)	NS
**EUS / MRI**	T3N0	84 (57.53%)	56 (40.58%)	NS
	T3N1	37 (25.34%)	40 (28.99%)	NS
	T3N2	4 (2.74%)	17 (12.32%)	< 0.05
	T4N0	4 (2.74%)	13 (9.42%)	< 0.05
	T4N1	5 (3.42%)	12 (8.70%)	NS
	T4N2	12 (8.22%)	0 (0.00%)	< 0.05
**Distance to anal verge (cm)** **EUS / MRI**	≤ 5	73 (50.00%)	58 (42.03%)	NS
	5.1–10	42 (28.77%)	57 (41.30%)	NS
	10.1–15	31 (21.23%)	23 (16.67%)	NS

Furthermore, the EUS and MRI staging revealed that Group A had a higher prevalence of advanced disease at baseline than Group B, with a more significant proportion of patients with T4N2 stage indicating more extensive tumour involvement and depth of invasion and nodal metastasis. Finally, the distance distribution to the anal verge varied between the two groups. While both groups had a predominance of patients with a distance of less than or equal to 5 cm, Group A had a higher proportion of such patients, accounting for 50% of the group (Table 1).

We observed notable differences in their respective outcomes after comparing the pathological results of two distinct treatment groups (Table 2).

**Table 2:** Pathology Results. The outcomes are measured regarding local recurrence, distant metastasis, downstaging of the tumour (T & N, T, N), pathological complete response (pCR), and three-year disease-free survival

Variable		Group A (Neoadjuvant chemotherapy) (*n* = 146)	Group B (Standard-of-care) (*n* = 138)	Relative Risk (95% CI)	*P* value
**Down Stage (T & N)**	Yes	118 (80.82%)	84 (60.87%)	2.040 (1.388 to 3.031)	< 0.001
	No	28 (19.18%)	54 (39.13%)		
**Down Stage (T)**	Yes	106 (72.60%)	79 (57.24%)	1.561 (1.130 to 2.171)	0.009
	No	40 (27.40%)	59 (42.75%)		
**Down Stage (N)**	Yes	80 (54.79%)	57 (41.3%)	1.298 (1.037 to 1.653)	0.025
	No	66 (45.21%)	81 (58.70%)		
**Pathological Complete Response (pCR)**	Yes	38 (26.03%)	21 (15.22%)	1.136 (1.018 to 1.299)	0.030
	No	108 (73.97%)	117 (84.78%)		

Patients in group A experienced a marked, statistically significant downstaging of tumour size (T) and lymph node (N) involvement, with a risk reduction of 2.040 times (95% CI 1.388 to 3.031) compared to group B. Specifically, 80.82% of patients in group A underwent downstaging of T and N, whereas only 60.87% of group B patients exhibited the same response. Furthermore, 72.60% of Group A patients displayed downstaging of T, in contrast to 57.25% of Group B patients (*p* = 0.009), and 54.79% of Group A patients achieved downstaging of N, compared to 41.30% of those in Group B (*p* = 0.025). These findings elucidate that those patients in group A exhibited a more favourable treatment response, with the treatment demonstrating greater efficacy in reducing tumour size and lymph node involvement compared to group B patients. Also, a noteworthy proportion of patients in group A, constituting 26.03%, attained a pathological complete response (pCR) in contrast to 15.22% in group B. The relative risk of achieving pCR was calculated as 1.136 (95% CI, 1.018 to 1.299), signifying those patients in Group A exhibited a higher likelihood of achieving pCR, with this contrast being statistically significant (*p* = 0.030).

**Fig. 2:**
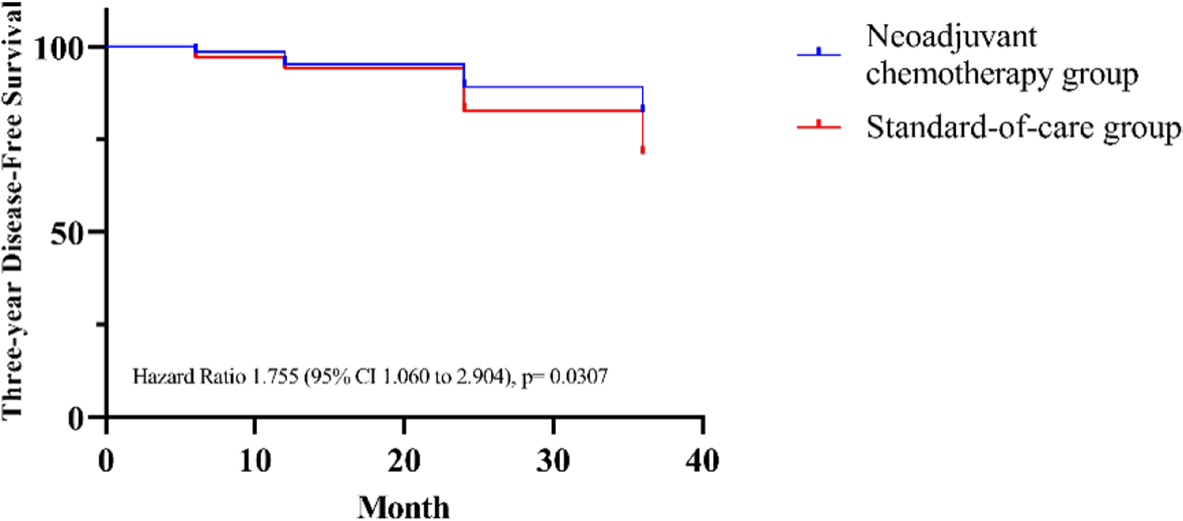
Kaplan–Meier estimates of 3-year disease-free survival

Notably, it was found that patients in group A had a significantly lower local cancer recurrence rate compared to those in group B (*P* = 0.025, 11.64% versus 21.74%, respectively). Within group A, 17 patients experienced local recurrence, with six at the primary site and 11 at non-primary sites. Similarly, in group B, 13 patients experienced local recurrence at the primary site and 17 at non-primary sites. Furthermore, the incidence of distant cancer metastasis exhibited a reduction, although it did not reach statistical significance (*p* = 0.143) in group A (8.90%) in comparison to group B patients (20.29%). These results indicate that patients in group A were less susceptible to cancer recurrence, with a risk reduction of 1.867 times (95% CI 1.091 to 3.220) for local recurrence and 1.709 (95% CI 0.9032 to 3.252) times for distance metastasis. Within group A, 82.19% of individuals remained free from the disease, while 17.81% experienced recurrence, encompassing local and distant manifestations. In contrast, group B exhibited a disease-free survival rate of 71.01%, with 28.99% encountering disease recurrence. The calculation of relative risk yielded a value of 0.8640, accompanied by a 95% CI ranging from 0.7530 to 0.9824 (p = 0.035). These findings strongly indicate that Group A participants exhibited a significantly lower risk of disease recurrence than Group B. On the other hand, group B has a higher risk of the recurrence of cancer (Hazard ratio 1.755, 95% CI 1.060 to 2.904 (*p* = 0.307) (Fig. 2).

We analysed adverse events in two groups of patients, focusing on grade 1–2 and severe (grades 3 and 4) adverse events across haematological, non-haematological, and biochemical categories. In the grade 1–2 adverse events category, both groups exhibited anaemia as the most prevalent adverse event in the haematological category, followed by thrombocytopenia and leukopenia. Febrile neutropenia was infrequently reported, with only one case documented in Group B. Notably, nausea/vomiting and peripheral sensory neuropathy emerged as the commonly reported non-haematological adverse events in both groups, while palmar-plantar erythrodysesthesia was the least frequently reported. In the biochemical category, both groups demonstrated mildly elevated levels of SGOT and SGPT compared to bilirubin and creatinine levels.

In the severe adverse events category, both groups reported cases of leukopenia and neutropenia, with Group B exhibiting slightly more incidents of leukopenia. At the same time, Group A had more cases of neutropenia. Notably, neither group reported occurrences of febrile neutropenia, thrombocytopenia, or anaemia in the severe adverse events category, nor did they report instances of nausea/vomiting, peripheral sensory neuropathy, palmar-plantar erythrodysesthesia, or constipation in the non-haematological sever complication category. In the biochemical category, one case of elevated SGPT was reported in both groups, with elevated bilirubin slightly more prevalent in Group B than Group A, although the variance was not statistically significant. Additionally, both groups reported two cases of elevated creatinine (Table 3). These results indicate that both treatment strategies possess a similar safety profile, with a comparable number of adverse events reported in each category. However, it is essential to note that the absence of severe adverse events does not necessarily imply that the treatments were well-tolerated, as patients may have experienced less severe but still impactful side effects.

**Table 3:** Adverse events. The adverse events were categorised into haematological, non-haematological, and biochemical. Each category was further divided into two severity grades: Grades 1 & 2 and Grades 3 & 4. NS, not significant; SGOT, Aspartate transaminase; SGPT, Alanine transaminase

		**Group A (Neoadjuvant chemotherapy) (*n* = 146)**	**Group B (Standard-of-care) (*n* = 138)**	***P* value**	**Group A (Neoadjuvant chemotherapy) (*n* = 146)**	**Group B (Standard-of-care) (*n* = 138)**	***P* value**
**Adverse events**		**Grades 1 & 2**			**Grades 3 & 4**		
**Haematological**	**Leukopenia**	58 (40%)	66 (48%)	NS	3 (2%)	5 (4%)	NS
	**Neutropenia**	38 (26%)	43 (31%)	NS	9 (6%)	15 (11%)	NS
	**Febrile neutropenia**	0 (0%)	1 (1%)	NS	0 (0%)	0 (0%)	NS
	**Thrombocytopenia**	41 (28%)	52 (37%)	NS	0 (0%)	0 (0%)	NS
	**Anaemia**	85 (58%)	94 (68%)	NS	0 (0%)	0 (0%)	NS
**Non-haematological**	**Nausea**	41 (28%)	42 (30%)	NS	2 (1%)	3 (2%)	NS
	**Vomiting**	8 (5%)	11 (8%)	NS	0 (0%)	0 (0%)	NS
	**Peripheral sensory neuropathy**	92 (63%)	88 (64%)	NS	17 (12%)	28 (20%)	NS
	**Palmar-plantar erythrodysesthesia**	11 (8%)	17 (12%)	NS	2 (1%)	5 (4%)	NS
	**Constipation**	15 (10%)	17 (12%)	NS	0 (0%)	0 (0%)	NS
**Biochemical**	**Elevated SGOT**	43 (29%)	46 (33%)	NS	2 (1%)	1 (1%)	NS
	**Elevated SGPT**	47 (32%)	45 (32%)	NS	1 (1%)	1 (1%)	NS
	**Elevated Bilirubin**	3 (2%)	4 (3%)	NS	0 (0%)	0 (0%)	NS
	**Elevated Creatinine**	14 (10%)	15 (11%)	NS	2 (1%)	2 (1%)	NS

## Discussion

Recent years have seen the emergence of numerous alternative approaches to the treatment of rectal cancer. Pilot studies and phase 2 randomised trials have shown that neoadjuvant chemotherapy, compared with adjuvant chemotherapy, not only does not compromise the efficacy of preoperative chemoradiotherapy but also offers a more tolerable toxicity profile. This increases drug concentration, enhances compliance with the treatment regimen, significantly improves disease-free survival and reduces local recurrence. While no significant differences in disease-free survival or overall survival have been observed, the promise of neoadjuvant chemotherapy is a beacon of hope in the fight against rectal cancer [17]. In this study, neoadjuvant chemotherapy (group A) significantly improves outcomes compared to surgery alone (group B) in treating locally advanced rectal cancer. Group A exhibits higher pathologic complete response (pCR) rates, overall down-staging, and tumour regression. Additionally, group A has fewer metastatic regional lymph nodes and lower local cancer recurrence rates. Although distant metastasis differences are not statistically significant, group A shows higher disease-free survival. These findings underscore neoadjuvant chemotherapy’s potential in managing locally advanced rectal cancer.

Recent randomised trials, particularly the RAPIDO, PROSPECT, and PRODIGE 23 trials, have yielded significant findings in the use of neoadjuvant chemotherapy in the treatment of rectal cancer RAPIDO [18], PROSPECT [19], and PRODIGE 23 [17]. Our research has shown that incorporating neoadjuvant chemotherapy alongside chemoradiation can significantly reduce local recurrence and potentially limit distant metastasis. This potential to improve disease-free survival could be a game-changer in developing effective treatment plans for cancer patients.

The RAPIDO trial showed that preoperative short-course radiation with CAPOX chemotherapy had lower disease-related treatment failure than preoperative chemoradiotherapy. However, these findings have been attributed to the lower rates of chemotherapy use in the chemoradiotherapy group in that trial. Additionally, the 5-year risk of local recurrence was high with short-course radiation, which may explain the limited adoption of this treatment strategy in North America [20]. However, our trial determined that adding the correct number and the proper neoadjuvant chemotherapy regimen improved therapeutic outcomes and specifically decreased local recurrence in three years.

The PROSPECT trial was aimed to compare the effectiveness of neoadjuvant FOLFOX, with selective use of pelvic chemoradiotherapy, to the current standard of neoadjuvant pelvic chemoradiotherapy alone. The trial was conducted for patients with clinically staged T2 node-positive, T3 node-negative, or T3 node-positive rectal cancer who were eligible for sphincter-sparing surgery. The trial results indicated that both treatment approaches demonstrated similar disease-free and overall survival rates. This suggests that neoadjuvant chemotherapy could be as effective as standard chemoradiation in selective patients. The results of our study confirmed this hypothesis. Our study observed that neoadjuvant FOLFOX combined with chemoradiation was not only non-inferior to the chemoradiation alone but also significantly improved disease-free survival in locally advanced rectal cancer compared to standard chemoradiation alone. This evidence could be observed in group A, where 17 patients experienced local recurrence, compared with 30 patients in group B, who experienced local recurrence. This positive outcome can be attributed to the addition of a potent neoadjuvant chemotherapy regimen to the standard chemoradiation in these high-risk patients. Notably, the incidence of local recurrence in the PROSPECT trial was less than 2% after five years, which is lower than in our previous trials. This disparity may be attributed to excluding T4 disease (T4 N0, T4 N +) and locally advanced tumours unsuitable for sphincter-sparing surgery in the PROSPECT trial.

In the PRODIGE 23 trial, which is similar to our study, patients with locally advanced rectal cancer who underwent neoadjuvant FOLFIRINOX treatment had notably extended periods of disease-free and metastasis-free survival than those who underwent standard chemoradiotherapy and adjuvant chemotherapy. However, the FOLFIRINOX approach is complex and may lead to heightened complications and intolerance in patients. To address this, we utilised a modified MFOLFOX6 as neoadjuvant chemotherapy for patients with stage T3 or T4 and N + rectal cancer before chemoradiation. The intervention group received six cycles of MFOLFOX6 before chemoradiotherapy surgery and six more courses of MFOLFOX6 after surgery. The standard-of-care group was given chemoradiotherapy followed by surgery and 12 courses of MFOLFOX6 chemotherapy. Our study demonstrated a notable increase in pathologic complete response, tumour and lymph node downstaging, and a significant reduction in 3-year local recurrence for the intervention group, compared to the standard-of-care group. Although insignificant, 3-year distant metastasis was also reduced in the intervention group. The use of MFOLFOX6 as induction chemotherapy did not compromise radiotherapy compliance or surgical quality and did not necessitate additional medical action.

Today, the treatment options for locally advanced rectal cancer have expanded, allowing for tailored treatment based on the tumour’s unique characteristics. This progress, such as successfully treating high microsatellite instability tumours with immunotherapy alone, is a significant step forward in cancer treatment [21]. On the other hand, until now, the addition of a monoclonal antibody to six cycles of a neoadjuvant fluoropyrimidine–oxaliplatin combination has not shown superior efficacy (e.g., the addition of bevacizumab or aflibercept substantially increased surgical complications in two trials [22, 23]. Adding cetuximab to neoadjuvant CAPOX followed by chemoradiotherapy did not increase pathological complete response or 5-year progression-free survival [24]. Hence, it can be inferred that investigating the utilisation of cost-effective chemotherapy drugs with high efficacy and minimal side effects presents a more favourable approach to enhancing therapeutic outcomes in patients with high-risk rectal cancer. However, it is crucial to exercise caution when considering additional treatments. Specifically, a careful assessment should be conducted for each tumour, considering its progression and each patient’s overall health status. The optimal approach should be selected based on these factors.

Consequently, a comprehensive evaluation was conducted to examine the effects of incorporating neoadjuvant FOLFOX chemotherapy alongside standard chemoradiation in treating high-risk rectal cancers in patients with suitable general conditions, including performance status and medical conditions. The rationale behind adopting FOLFOX was to mitigate complications, as it is associated with a well-defined and limited profile of adverse effects while concurrently delivering substantial therapeutic benefits. One notable strength of our study was the implementation of well-defined guidelines for administering neoadjuvant and adjuvant chemotherapy. These guidelines were instrumental in ensuring that the duration of chemotherapy, spanning six months, was consistent across both study groups.

In contrast to the PRODIGE23 trial, we did not encounter the need to modify the treatment regimen during the adjuvant phase or introduce a different regimen at this stage. This approach effectively minimised the potential for significant methodological bias in our study. A pivotal aspect of treatment in our study involved altering chemotherapy administration timing. Specifically, half of the chemotherapy courses were shifted from the adjuvant to the perioperative setting. This modification yielded remarkable results in our study, including definite outcome improvement and relative reduction of complications. The observed substantial progress can be attributed exclusively to the therapeutic effectiveness of the drugs administered during the neoadjuvant phase. This favourable outcome can be ascribed to the absence of any structural alterations or vascular damage during this specific stage of the treatment process.

The toleration of MFOLFOX6 chemotherapy in this study was good, and only two patients from one hundred forty-eight group A patients could not complete their chemotherapy, which was better than adjuvant chemotherapy in 4 patients who could not complete their whole chemotherapy courses. Also, upfront use of MFOLFOX6 did not increase surgical morbidity or adversely affect compliance with subsequent adjuvant chemotherapy. Because of the high rate of pathological complete response in this study by adding neoadjuvant chemotherapy, future studies should also evaluate more patients and probably more effective chemotherapy regimens to achieve more pathologic complete response and eliminate high-risk and unnecessary treatment modalities like surgery in this situation. These data logically indicate the possibility of risk-adapted therapy and decreasing significant and permanent side effects by modifying treatment modalities. Further evaluation of non-operative management and organ-preserving strategies in selected patients, even in locally advanced situations following neoadjuvant chemotherapy (such as the GRECCAR12 trial [25]), could be possible.

### Study limitations

An identified limitation of this investigation is the absence of a centralised review of study results, including physical exams, lab, pathology, and imaging at the participating centres. The decentralised approach may have introduced data collection, interpretation, and reporting variability, potentially impacting our findings’ robustness and coherence. It is essential to note that these results should be interpreted in the context of the study design and patient population. For example, the study population consisted of patients with locally advanced rectal cancer treated at a single institution, which may limit the generalizability of the findings. Other factors such as age, EUS staging, and distance distribution to the anal verge can also affect surgical outcomes and postoperative complications. More research is needed to confirm these findings and determine the most appropriate treatment approach for individuals with rectal cancer. Furthermore, the initial difference between groups A and B at the start of the trial was another limitation in this study. While this difference falls within acceptable bounds for a randomised controlled trial, it is still noticeable. We cautiously interpreted our conclusions, recognising the potential impact of these initial disparities on observed effects. We conducted sensitivity analyses and adjusted for baseline variables to address this limitation, reaffirming our findings’ strength. Looking ahead, we recommend further exploration of the impact of baseline differences on treatment outcomes. This presents exciting possibilities for future studies to consider alternative randomisation techniques or covariate adjustment methods to enhance the validity of future randomised controlled trials.

## Conclusion

In conclusion, our study findings indicate that neoadjuvant chemotherapy with MFOLFOX6 followed by chemoradiotherapy, surgery, and adjuvant chemotherapy is a promising treatment for patients with locally advanced rectal cancer. This treatment approach enhances pathologically complete response and reduces the T & N staging, resulting in better patient outcomes. Furthermore, the neoadjuvant chemotherapy group showed significantly improved disease-free survival without substantially increasing toxicity compared to the adjuvant chemotherapy group. This evidence indicates that the perioperative chemotherapy approach is more effective and better tolerated than adjuvant chemotherapy. These results are significant and robust enough to change clinical practice guidelines.

## Data Availability

The data generated and/or analysed during the current study are not publicly available but are available from the corresponding author who organised the study.

## References

[1] Siegel RL, Wagle NS, Cercek A, Smith RA, Jemal A. Colorectal cancer statistics, 2023. CA Cancer J Clin. 2023;73(3):233–54. 36856579 10.3322/caac.21772

[2] Franke AJ, Parekh H, Starr JS, Tan SA, Iqbal A, George TJ Jr. Total Neoadjuvant Therapy: A Shifting Paradigm in Locally Advanced Rectal Cancer Management. Clin Colorectal Cancer. 2018;17(1):1–12. 28803718 10.1016/j.clcc.2017.06.008

[3] Hull R, Francies FZ, Oyomno M, Dlamini Z. Colorectal Cancer Genetics, Incidence and Risk Factors: In Search for Targeted Therapies. Cancer Manag Res. 2020;12:9869–82. 33116845 10.2147/CMAR.S251223 PMC7553623

[4] Rawla P, Sunkara T, Barsouk A. Epidemiology of colorectal cancer: incidence, mortality, survival, and risk factors. Prz Gastroenterol. 2019;14(2):89–103. 31616522 10.5114/pg.2018.81072 PMC6791134

[5] Huang CM, Huang MY, Ma CJ, Yeh Y, Tsai HL, Huang CW, et al. Neoadjuvant FOLFOX chemotherapy combined with radiotherapy followed by radical resection in patients with locally advanced colon cancer. Radiat Oncol. 2017;12(1):48. 28270172 10.1186/s13014-017-0790-3 PMC5341372

[6] Hav M, Libbrecht L, Ferdinande L, Geboes K, Pattyn P, Cuvelier CA. Pathologic Assessment of Rectal Carcinoma after Neoadjuvant Radio(chemo)therapy: Prognostic Implications. Biomed Res Int. 2015;2015: 574540. 26509160 10.1155/2015/574540 PMC4609786

[7] Kapiteijn E, Marijnen CAM, Nagtegaal ID, Putter H, Steup WH, Wiggers T, et al. Preoperative Radiotherapy Combined with Total Mesorectal Excision for Resectable Rectal Cancer. N Engl J Med. 2001;345(9):638–46. 11547717 10.1056/NEJMoa010580

[8] Zwart WH, Hotca A, Hospers GAP, Goodman KA, Garcia-Aguilar J. The Multimodal Management of Locally Advanced Rectal Cancer: Making Sense of the New Data. Am Soc Clin Oncol Educ Book. 2022;42:264–77. 10.1200/EDBK_351411 35561302

[9] Martin JD, Seano G, Jain RK. Normalizing Function of Tumor Vessels: Progress, Opportunities, and Challenges. Annu Rev Physiol. 2019;81:505–34. 30742782 10.1146/annurev-physiol-020518-114700 PMC6571025

[10] Conde-Muíño R, Cuadros M, Zambudio N, Segura-Jiménez I, Cano C, Palma P. Predictive Biomarkers to Chemoradiation in Locally Advanced Rectal Cancer. Biomed Res Int. 2015;2015: 921435. 26504848 10.1155/2015/921435 PMC4609421

[11] Patel SA, Ryan DP, Hong TS. Combined Modality Therapy for Rectal Cancer. Cancer J. 2016;22(3):211–7. 27341601 10.1097/PPO.0000000000000193

[12] Yoon WH, Kim HJ, Kim CH, Joo JK, Kim YJ, Kim HR. Oncologic impact of pathologic response on clinical outcome after preoperative chemoradiotherapy in locally advanced rectal cancer. Ann Surg Treat Res. 2015;88(1):15–20. 25553320 10.4174/astr.2015.88.1.15 PMC4279989

[13] Benson AB, Venook AP, Al-Hawary MM, Azad N, Chen YJ, Ciombor KK, et al. Rectal Cancer, Version 2.2022, NCCN Clinical Practice Guidelines in Oncology. J Natl Compr Canc Netw. 2022;20(10):1139–67. 36240850 10.6004/jnccn.2022.0051

[14] Wu HQ, Li J, Miao JD, Li JW. Is Total Neoadjuvant Treatment Beneficial for Locally Advanced Rectal Cancer? A Meta-Analysis of Randomized Controlled Trials. Oncology. 2024;102(5):399–413. 10.1159/000534815. 10.1159/000534815 37926087

[15] Schulz KF, Altman DG, Moher D, the CG. CONSORT 2010 Statement: updated guidelines for reporting parallel group randomised trials. BMC Med. 2010;8(1):18. 20334633 10.1186/1741-7015-8-18 PMC2860339

[16] Daniel Enderlein G., Wayne W. Biostatistics — A Foundations for Analysis in the Health Sciences. Wiley & Sons, New York—Chichester—Brisbane—Toronto—Singapore, 6th ed. 1995, 780 S., £58.—, ISBN 0–471–58852‐0 (cloth). Biom J. 2007;37(6):744.

[17] Conroy T, Etienne P-L, Rio E, Evesque L, Mesgouez-Nebout N, Vendrely V, et al. Total neoadjuvant therapy with mFOLFIRINOX versus preoperative chemoradiation in patients with locally advanced rectal cancer: 7-year results of PRODIGE 23 phase III trial, a UNICANCER GI trial. J Clin Oncol. 2023;41(17_suppl):LBA3504-LBA. 10.1200/JCO.2023.41.17_suppl.LBA3504 38986769

[18] Bahadoer RR, Dijkstra EA, van Etten B, Marijnen CAM, Putter H, Kranenbarg EM, et al. Short-course radiotherapy followed by chemotherapy before total mesorectal excision (TME) versus preoperative chemoradiotherapy, TME, and optional adjuvant chemotherapy in locally advanced rectal cancer (RAPIDO): a randomised, open-label, phase 3 trial. Lancet Oncol. 2021;22(1):29–42. 33301740 10.1016/S1470-2045(20)30555-6

[19] Schrag D, Shi Q, Weiser MR, Gollub MJ, Saltz LB, Musher BL, et al. Preoperative Treatment of Locally Advanced Rectal Cancer. N Engl J Med. 2023;389(4):322–34. 37272534 10.1056/NEJMoa2303269 PMC10775881

[20] Dijkstra EA, Nilsson PJ, Hospers GAP, Bahadoer RR, Meershoek-Klein Kranenbarg E, Roodvoets AGH, et al. Locoregional Failure During and After Short-course Radiotherapy Followed by Chemotherapy and Surgery Compared With Long-course Chemoradiotherapy and Surgery: A 5-Year Follow-up of the RAPIDO Trial. Ann Surg. 2023;278(4):e766–72. 36661037 10.1097/SLA.0000000000005799 PMC10481913

[21] Cercek A, Lumish M, Sinopoli J, Weiss J, Shia J, Lamendola-Essel M, et al. PD-1 Blockade in Mismatch Repair-Deficient, Locally Advanced Rectal Cancer. N Engl J Med. 2022;386(25):2363–76. 35660797 10.1056/NEJMoa2201445 PMC9492301

[22] Borg C, Mantion G, Boudghène F, Mornex F, Ghiringhelli F, Adenis A, et al. Efficacy and Safety of Two Neoadjuvant Strategies With Bevacizumab in MRI-Defined Locally Advanced T3 Resectable Rectal Cancer: Final Results of a Randomized, Noncomparative Phase 2 INOVA Study. Clin Colorectal Cancer. 2019;18(3):200-8.e1. 31311761 10.1016/j.clcc.2019.04.006

[23] Fernández-Martos C, Pericay C, Losa F, García-Carbonero R, Layos L, Rodríguez-Salas N, et al. Effect of Aflibercept Plus Modified FOLFOX6 Induction Chemotherapy Before Standard Chemoradiotherapy and Surgery in Patients With High-Risk Rectal Adenocarcinoma: The GEMCAD 1402 Randomized Clinical Trial. JAMA Oncol. 2019;5(11):1566–73. 31465088 10.1001/jamaoncol.2019.2294 PMC6865228

[24] Leichman CG, McDonough SL, Smalley SR, Billingsley KG, Lenz HJ, Beldner MA, et al. Cetuximab Combined With Induction Oxaliplatin and Capecitabine, Followed by Neoadjuvant Chemoradiation for Locally Advanced Rectal Cancer: SWOG 0713. Clin Colorectal Cancer. 2018;17(1):e121–5. 29233486 10.1016/j.clcc.2017.10.008 PMC6598683

[25] Rullier E, Vendrely V, Asselineau J, Rouanet P, Tuech JJ, Valverde A, et al. Organ preservation with chemoradiotherapy plus local excision for rectal cancer: 5-year results of the GRECCAR 2 randomised trial. Lancet Gastroenterol Hepatol. 2020;5(5):465–74. 32043980 10.1016/S2468-1253(19)30410-8

